# Applications of CRISPR-Cas9 mediated genome engineering

**DOI:** 10.1186/s40779-015-0038-1

**Published:** 2015-05-09

**Authors:** Xiao Yang

**Affiliations:** State Key Laboratory of Proteomics, Collaborative Innovation Center for Cardiovascular Disorders, Genetic Laboratory of Development and Disease, Institute of Biotechnology, Beijing, 100071 China

**Keywords:** CRISPR-Cas9, Genome editing, Functional genomic screening, Gene therapy, Animal model

## Abstract

Targeted mutagenesis based on homologous recombination has been a powerful tool for understanding the mechanisms underlying development, normal physiology, and disease. A recent breakthrough in genome engineering technology based on the class of RNA-guided endonucleases, such as clustered regularly interspaced short palindromic repeats (CRISPR)-associated Cas9, is further revolutionizing biology and medical studies. The simplicity of the CRISPR-Cas9 system has enabled its widespread applications in generating germline animal models, somatic genome engineering, and functional genomic screening and in treating genetic and infectious diseases. This technology will likely be used in all fields of biomedicine, ranging from basic research to human gene therapy.

## Introduction

In the post-genome era, further defining the biological function of genome sequences and their transcripts in development and disease is a huge challenge. Targeted mutagenesis in whole animal is a powerful method to elucidate gene function in a physiological setting. The development of gene targeting, in which specific gene modification can be introduced into the animal genome through homologous recombination and embryonic stem cell technology, has revolutionized biological and biomedical studies [1]. Genetically modified animals, especially gene knockout mice, have been valuable as human disease models. The second generation of gene targeting, conditional gene targeting based on site-specific recombinases, primarily the Cre-LoxP system, enables the creation of animals carrying targeted mutations in specific cell types or in an inducible manner [2]. A new generation of genome engineering technologies based on the class of RNA-guided endonucleases, such as clustered regularly interspaced short palindromic repeats (CRISPR)-associated Cas9, and their rapid applications are now bringing a further revolution in biology and medicine [3,4]. This review will focus on the recent progress of the applications of CRISPR-Cas9-mediated genome engineering.

## Review

### CRISPR-Cas9 technology

**Figure 1:**
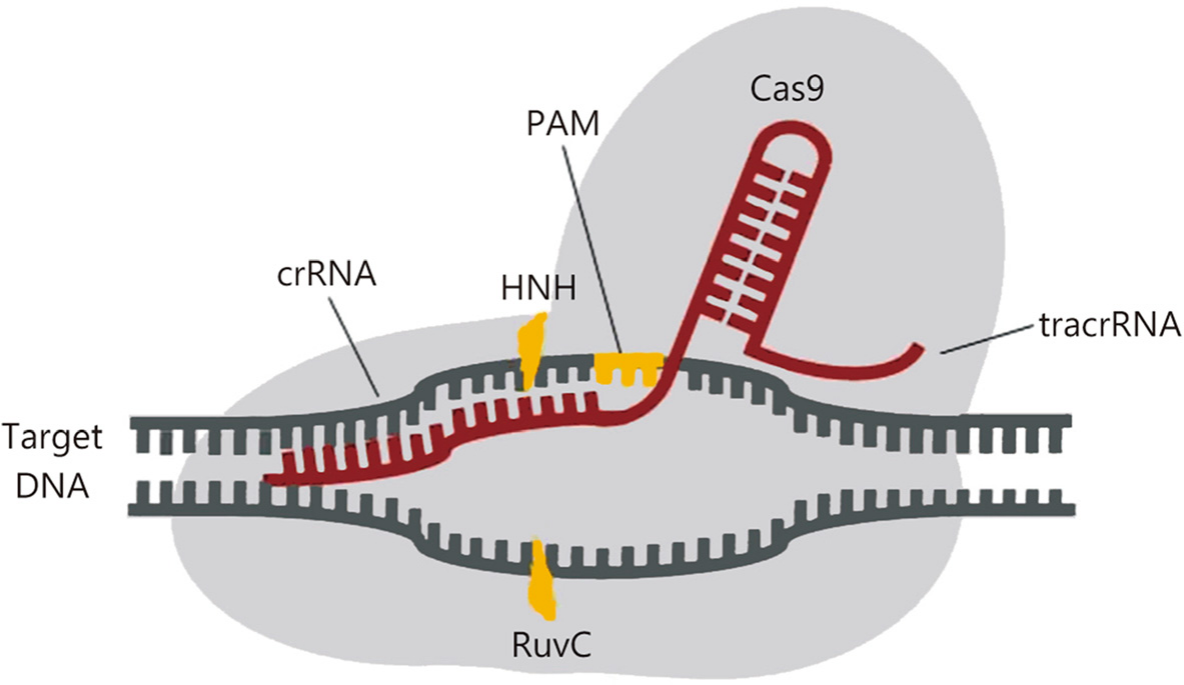
The CRISPR-Cas system. The CRISPR-associated endonuclease Cas9 could target specific DNA loci and make double-strand breaks under the guidance of the tracrRNAs:crRNAs duplex. The tracrRNA:crRNA duplex directs Cas9 to use two distinct active sites, RuvC and HNH, and cleave the target DNA complementary to the crRNA, which has an adjacent protospacer-adjacent motif (PAM).

The CRISPR-Cas9 technology is developed from type II CRISPR-Cas systems, by which bacteria degrade targeted nucleic acids [5,6]. CRISPRs are characterized features of the genomes of most bacteriophage-resistant Bacteria and Archaea. Cas9, a CRISPR-associated endonuclease with putative nuclease and helicase domains, can be localized to specific DNA loci and make double-strand breaks under the guidance of the trans-activating CRISPR RNA (tracrRNA):CRISPR RNA (crRNA) duplex (Figure 1) [7-9]. tracrRNA has been shown to help RNA to direct the maturation of crRNAs by host RNase III and the CRISPR-associated Csn1 protein, protecting *S. pyogenes* against prophage-derived DNA [9]. Further studies have shown that the tracrRNA:crRNA duplex directs the CRISPR-associated protein Cas9 from *S. thermophilus* or *S. pyogenes* to use two distinct active sites, RuvC and HNH, and cleave the two strands in the target DNA, which is complementary to the crRNA [10,11]. The dual tracrRNA:crRNA was further developed as a single-guide RNA (sgRNA) for genome engineering and contains the 5′ end 20-nucleotide sequence determining the DNA target site according to Watson-Crick base pairing and 3′ end double-stranded structure binding Cas9 [10]. The sgRNA could direct CRISPR-Cas9 to any target DNA sequence with an adjacent protospacer-adjacent motif (PAM) by changing the guide RNA sequences [12-14]. Expectedly, the CRISPR-Cas9 has been shown to be an effective approach for genome editing in human cells [15-17]. The simplicity of the CRISPR-Cas9 system has enabled widespread applications of this system for efficient genome engineering in various species.

A major concern in the application of CRISPR-Cas9 technology is the targeting specificity of Cas9 nucleases. A number of studies have shown that Cas9 could tolerate some mismatches between the guide RNA and its complementary target DNA sequence, causing potential off-site targeting [10,15,18]. Off-site targeting is largely affected by the number, position, distribution of mismatches and Cas9 concentration, and may be a major concern in CRISPR/Cas9-medicated genome engineering [15,19-22]. Genome-wide Cas9 cutting specificity has been studied by high-throughput Cas9-based chromatin immunoprecipitation sequencing (ChIP-seq) analysis [23-25], and more unbiased experiments are required for elucidating the mechanisms of Cas9 binding and cleavage specificity. Further optimizations using the double nicking strategy and shorter sgRNAs have significantly reduced off-target activity [26,27]. Several groups have provided tools for designing highly active sgRNAs and minimizing off-site targeting (http://tools.genome-engineering.org, http://zifit.partners.org, and www.e-crisp.org) [28-32].

### CRISPR-Cas9 in the generation of animal models

Gene targeting based on homologous recombination and embryonic stem cells has been used as the typical approach for animal genome modification, which has played indispensable roles in making a causal link between genomic mutations and phenotypes during development and in disease. However, gene targeting has limited applications in some organisms due to time-consuming procedures and the lack of available embryonic stem cells. Many recent studies have shown that CRISPR-Cas9 technology could be used for rapidly generating targeted genome modifications in the germ lines of various model organisms [33-47], which will significantly advance the functional genomics.

Microinjection of Cas9-encoding mRNA and customizable sgRNA into one-cell stage zebrafish embryos is able to efficiently modify the target genes *in vivo* in a simple, rapid and scalable manner [33,34]. Co-injection of Cas9 mRNA and sgRNAs targeting different genes into mouse zygotes generates mutant mice with biallelic mutations, confirming that CRISPR/Cas-mediated gene editing could be used for the simultaneous disruption of multiple genes with high efficiency [35]. Gene knockin mice carrying precise point mutations of two genes can be obtained by co-injection of Cas9 mRNA/sgRNAs together with mutant oligos [36]. The following study demonstrates that reporter and conditional mutant mice can also be generated in one step by co-injecting mouse zygotes with Cas9 mRNA and different sgRNAs, as well as DNA vectors of different sizes. Additionally, mice with the predicted deletions have been generated using sgRNAs targeting two separate sites in the gene [35]. Multiplexed activation of endogenous genes can be achieved by injecting a two-component transcriptional activator including a nuclease-dead Cas9 protein fused with a transcriptional activation domain and sgRNAs targeting gene promoters [37]. These previous studies have demonstrated that CRISPR-Cas9 technology can be used for efficient one-step generation of various sophisticated mutant mice, including mice carrying gene insertions, deletions, conditional alleles and endogenous reporters at different loci. A recently established Cre-dependent Cas9 knockin mouse may further facilitate the generation of genetic modified mutant mice by simply injecting sgRNA [38].

CRISPR-Cas9 technology has been used for efficient genome engineering in many other model organisms, including *Drosophila* [39,40], *Caenorhabditis elegans* [41], *Axolotl* [42], *Xenopus tropicalis* [43,44], rat [45] and pig [46]. Significantly, the CRISPR-Cas9 system has been shown to be an efficient and reliable approach for targeted modification of cynomolgus monkey genomes [47]. The application of CRISPR-Cas9 technology for genome editing in a wide range of organisms will promote our understanding of development and disease and help develop animal models and therapeutic strategies for human diseases.

### CRISPR-Cas9 in somatic genome editing

Rapid progress in genome engineering based on the CRISPR-Cas9 system enables fast functional characterization of putative disease genes in various mouse models *via* somatic genome editing [48-50]. A CRISPR plasmid DNA expressing Cas9 and sgRNAs can be delivered to the liver through hydrodynamic injection, and CRISPR-mediated Pten mutation with or without p53 mutation phenocopies the effects of PTEN and p53 gene knockout using Cre-LoxP technology [48]. Previous studies have also shown that an activated mutant β-catenin gene could be delivered into hepatocytes by co-injection of Cas9 plasmids expressing sgRNAs targeting the β-catenin gene and a DNA oligonucleotide donor carrying β-catenin activating point mutations [48]. This previous study demonstrated that the CRISPR-Cas system could be used for directly mutating tumor suppressor genes and oncogenes in somatic tissues, providing a new approach for developing new types of disease models. The CRISPR-Cas9 system has also been used to induce a specific chromosomal rearrangement, the Eml4-Alk inversion, in somatic cells of adult animals to generate a mouse model of Eml4-Alk-driven lung cancer [49]. The resulting tumors exhibit the typical histopathological and molecular features of ALK(+) human non-small cell lung cancer (NSCLC), which is sensitive to ALK inhibitors [49]. Interestingly, using a lentiviral-based delivery system, a recent study demonstrated that CRISPR-induced genome editing of tumor suppressor genes together with Cre-dependent somatic activation of oncogenic Kras(G12D) causes lung adenocarcinomas with different histopathological and molecular features [50]. Using the Cas9 gene knockin mice, lung adenocarcinoma models can be generated by simultaneously introducing a single AAV vector carrying loss-of-function mutations in p53, Lkb1 and Kras(G12D) mutations in the lung [38], suggesting that Cas9 gene knockin mice could be widely used for somatic genome editing. The rapid somatic genome engineering approach will greatly help to systematically identify critical genes underlying disease initiation and progression in many well-established disease mouse models.

### CRISPR-Cas9 in functional genomic screening

Functional genomic screening is largely used for identifying the essential genes for a specific cellular process. RNA interference (RNAi) [51] has been dominantly applied for genome-wide screening; however, the off-target effects of RNAi has limited its applications [52-54]. In addition, RNAi could not be used for silencing RNAs located in nucleus. The CRISPR-Cas9 system has been successfully used in various genome-scale loss of function screening [55-58]. Using a genome-scale lentiviral sgRNA library, all expected genes of the DNA mismatch repair pathway have been identified in screening for resistance to the nucleotide analog 6-thioguanine, and numerous genes corresponding to fundamental processes have been obtained with a negative selection screening for essential genes [55]. A genome-scale CRISPR-Cas9 knockout (GeCKO) library has been developed and successfully used for screening genes essential for cell viability in cancer and pluripotent stem cells and for genes associated with the resistance to vemurafenib, a drug for late-stage melanoma [56]. A CRISPR-Cas-based knockout library has been applied to identify the host genes mediating the cellular responses to anthrax and diphtheria toxins [57]. A recent study has shown that saturation editing of genomic regions could be achieved by coupling CRISPR-Cas9 technology with multiplex homology-directed repair using a complex library of donor templates, facilitating high-resolution functional screening of both cis-regulatory elements and trans-acting factors in the genome [58].

A series of studies has demonstrated that CRISPR-mediated repression (CRISPRi) and CRISPR-mediated activation (CRISPRa) are powerful tools for functional genomic screening. A CRISPRi system consisting of a catalytically inactive Cas9 and a guide RNA has been shown to specifically and efficiently repress the transcription of target genes in *Escherichia coli* and mammalian cells [59,60], whereas a catalytically inactive Cas9 fused with a transcriptional activation domain has been used to activate the expression of specific endogenous genes [61-63]. Genome-scale CRISPRi and CRISPRa libraries that specifically target transcriptional repressors or activators to endogenous genes have been successfully used for screening essential genes for growth, tumor suppression, differentiation regulation, and cellular sensitivity to a cholera-diphtheria toxin, suggesting that CRISPRi and CRISPRa are valuable tools for mapping complex pathways [64]. A very recent study has shown that CRISPR-Cas9 complexes with synergistic activation mediators are able to achieve robust, single sgRNA-mediated gene upregulation at endogenous genomic loci. When used with an sgRNA library, the engineered Cas9 activation complexes can activate multiple genes simultaneously, upregulate long intergenic non-coding RNA transcripts and identify genes conferring resistance to a BRAF inhibitor through a genome-wide dCas9-based transcription activation screening in a melanoma model [65]. These results demonstrate that CRISPR-Cas9 technology can be a promising functional genomic screening tool for discovering essential genes in various biological processes.

### CRISPR-Cas9 in correction of genetic disorders

One of the most exciting applications of the CRISPR-Cas9 is the possibility of curing genetic diseases. The CRISPR-Cas9 system has been shown to efficiently correct a dominant Crygc gene mutation in a cataracts mouse model by co-injecting Cas9 mRNA and sgRNA targeting the mutant Crygc allele into zygotes [66]. A very recent study has shown that the CRISPR-Cas9 system can be used to modify an EGFP transgene or the endogenous Crygc gene in spermatogonial stem cells (SSCs). The modified SSCs carrying a corrected Crygc mutation can undergo spermatogenesis and produce offspring with the corrected phenotype at an efficiency of 100% [67]. The injection of Cas9, sgRNA, and homology-directed repair template into mouse zygotes has been shown to correct the dystrophin gene mutation responsible for muscular dystrophy in the germ line and prevent the development of muscular dystrophy in mutant mice [68]. Interestingly, a similar strategy using the CRISPR-Cas9 technology has successfully corrected the cystic fibrosis transmembrane conductor receptor (CFTR) locus by homologous recombination in cultured intestinal stem cells of cystic fibrosis human patients [69], demonstrating that primary adult stem cells derived from patients with a single-gene hereditary defect could be corrected by CRISPR/Cas9 mediated homologous recombination, suggesting a promising strategy for gene therapy in human patients.

### CRISPR-Cas9 in the treatment of infectious diseases

Considering that the CRISPR-Cas system originally functions as an antiviral adaptive immune system in bacteria, this system could be used for treating infectious diseases by eradicating pathogen genomes from infected individuals. Recently, studies have shown that the CRISPR-Cas9 system can eliminate the HIV-1 genome and prevent new HIV infection [70,71]. When transfected into HIV-1 provirus-integrated human cells, an sgRNA expression vector targeting the long terminal repeats (LTR) of HIV-1 efficiently cleaves and mutates LTR target sites and suppresses LTR-driven viral gene expression. In addition, this system has been shown to delete viral genes from the host cell chromosome [70]. The high specificity of Cas9/sgRNAs in editing the HIV-1 target genome has also been recently demonstrated [71]. Cas9/sgRNAs efficiently inactivate HIV gene expression and replication in latently infected cells, including microglial, promonocytic and T cells. Significantly, Cas9/sgRNA mediated genome editing has been shown to immunize cells to prevent HIV-1 infection [71]. These results indicate that the CRISPR-Cas9 technology can serve as a potential tool for clinical applications to cure infectious diseases.

## Conclusions and perspectives

**Figure 2:**
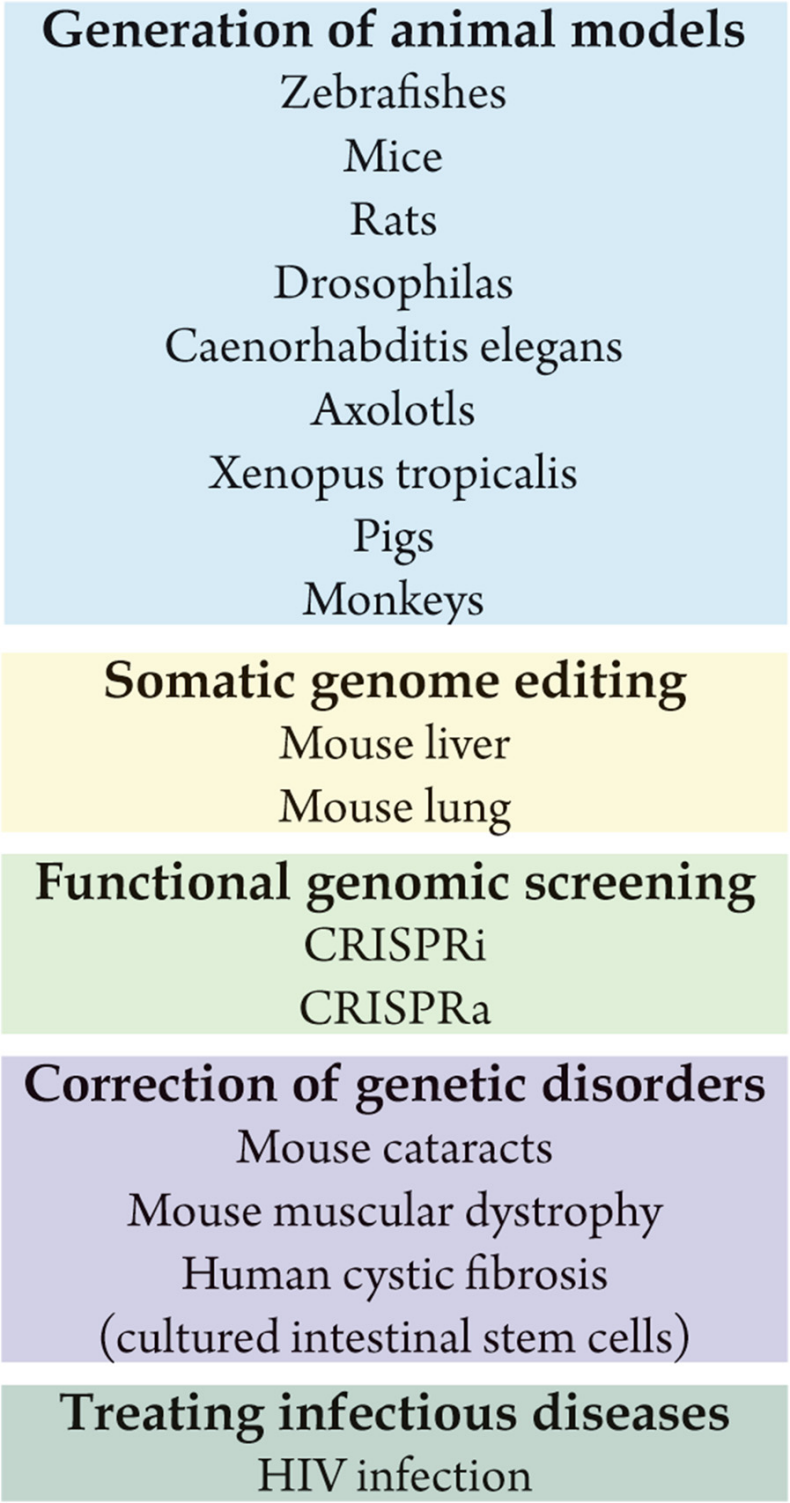
Applications of CRISPR-Cas9 mediated genome engineering.

The CRISPR-Cas9 technology, an efficient, inexpensive, fast-to-design, and easy-to-use genomic editing tool, has been rapidly applied in many fields, ranging from basic biology to translational medicine (Figure 2). The innovative applications of the CRISPR-Cas9 system will accelerate our understanding of the mechanisms underlying development, physiology and disease. The CRISPR-Cas9 technology will also accelerate research related to military medical sciences. Genome-wide functional screening will characterize essential genes that regulate host defense against pathogens. For example, a CRISPR-Cas9 based screening identified the host genes controlling the cellular responses to anthrax, diphtheria toxins and cholera-diphtheria toxins, providing new mechanisms of pathogen toxicity [53,55]. CRISPR-Cas9-based rapid generation of targeted model organisms will significantly boost the understanding of the mechanisms of adaption to an extreme or specific environment. A recent study using PAXX(-/-) cells generated by CRISPR-Cas9 demonstrated that the PAXX gene plays a role in DNA double-strand break repair and cell survival in response to ionizing radiation [72]. In the immediate future, the use of the CRISPR-Cas9 system will revolutionize military medical sciences and advance the fundamental knowledge of anti-pathogen defense, radiation protection, tissue homeostasis. Cas9/sgRNA mediated genome editing provides new therapeutic strategies for infectious diseases, wound healing and tissue regeneration.

## Abbreviations

CFTR: Cystic fibrosis transmembrane conductor receptor
ChIP-seq: Chromatin immunoprecipitation sequencing
CRISPR: Clustered regularly interspaced short palindromic repeats
CRISPRa: CRISPR-mediated activation
CRISPRi: CRISPR-mediated repressing
crRNAs: CRISPR RNAs
GeCKO: Genome-scale CRISPR-Cas9 knockout
LTR: Long terminal repeats
NSCLC: Non-small cell lung cancer
PAM: Protospacer-adjacent motif
RNAi: RNA interference
sgRNA: Single guide RNA
SSCs: Spermatogonial stem cells
tracrRNAs: Trans-activating CRISPR RNAs
